# Risk of hepatitis B virus reactivation and its effect on survival in advanced hepatocellular carcinoma patients treated with hepatic arterial infusion chemotherapy and lenvatinib plus programmed death receptor-1 inhibitors

**DOI:** 10.3389/fcimb.2024.1336619

**Published:** 2024-02-13

**Authors:** Zhenyun Yang, Renguo Guan, Yizhen Fu, Dandan Hu, Zhongguo Zhou, Minshan Chen, Yaojun Zhang

**Affiliations:** ^1^ Department of Liver Surgery, Sun Yat-sen University Cancer Center, Guangzhou, Guangdong, China; ^2^ Collaborative Innovation Center for Cancer Medicine, State Key Laboratory of Oncology in South China, Sun Yat-Sen University Cancer Center, Guangzhou, Guangdong, China; ^3^ Guangdong Provnvial Clinical Research Center for Cancer, Sun Yat-Sen University Cancer Center, Guangzhou, Guangdong, China

**Keywords:** HBV reactivation, hepatocellular carcinoma, hepatic arterial infusion chemotherapy, Lenvatinib, programmed cell death receptor-1 inhibitor, survival

## Abstract

**Background:**

Hepatitis B virus (HBV) reactivation is a common complication in hepatocellular carcinoma (HCC) patients treated with chemotherapy or immunotherapy. This study aimed to evaluate the risk of HBV reactivation and its effect on survival in HCC patients treated with HAIC and lenvatinib plus PD1s.

**Methods:**

We retrospectively collected the data of 213 HBV-related HCC patients who underwent HAIC and lenvatinib plus PD1s treatment between June 2019 to June 2022 at Sun Yat-sen University, China. The primary outcome was the risk of HBV reactivation. The secondary outcomes were overall survival (OS), progression−free survival (PFS), and treatment−related adverse events.

**Results:**

Sixteen patients (7.5%) occurred HBV reactivation in our study. The incidence of HBV reactivation was 5% in patients with antiviral prophylaxis and 21.9% in patients without antiviral prophylaxis, respectively. The logistic regression model indicated that for HBV reactivation, lack of antiviral prophylaxis (*P*=0.003) and tumor diameter (*P*=0.036) were independent risk factors. The OS and PFS were significantly shorter in the HBV reactivation group than the non-reactivation group (*P*=0.0023 and *P*=0.00073, respectively). The number of AEs was more in HBV reactivation group than the non-reactivation group, especially hepatic AEs.

**Conclusion:**

HBV reactivation may occur in HCC patients treated with HAIC and lenvatinib plus PD1s. Patients with HBV reactivation had shorter survival time compared with non-reactivation. Therefore, HBV-related HCC patients should undergo antiviral therapy and HBV-DNA monitoring before and during the combination treatment.

## Introduction

Hepatocellular carcinoma (HCC) is one of the most commonly occurring malignancies worldwide and among the top three leading causes of cancer-related deaths (Forner et al., 2018). Considering the current situation, more than a million new HCC patients and more than a million HCC-related deaths are estimated to occur in 2040 (Rumgay et al., 2022). Recently, systemic therapy has gradually become the mainstream treatment for advanced HCC. According to the IMbrave150 trial, atezolizumab–bevacizumab combination therapy is now recommended as the first-line therapeutic strategy for unresectable HCC (Finn et al., 2020). Furthermore, tyrosine kinase inhibitors (TKIs), such as levatinib, are widely used in advanced HCC patients (Heimbach et al., 2018). In addition to immunotherapy and targeted therapy, hepatic arterial infusion chemotherapy (HAIC) with fluorouracil, leucovorin, and oxaliplatin (FOLFOX) has emerged gradually over the past years. According to some clinical trials, HAIC could prolong the survival of advanced HCC patients better than sorafenib and conventional transarterial chemoembolization (TACE) (Li et al., 2022; Lyu et al., 2022; Lyu et al., 2018). Moreover, HAIC has been recommended for managing advanced HCC in Asia (Kudo et al., 2021; Zhou et al., 2020). A retrospective research clarified that HAIC could enhance the efficacy of lenvatinib and programmed death receptor-1 inhibitors (PD1s) (Fu et al., 2023).

As a dominant risk factor for HCC, hepatitis B virus (HBV) infection occurs in approximately 54% of HCC patients (European Association for the Study of the Liver, 2018; Chan et al., 2016). Particularly in China, more than 80% of the HCC cases are associated with HBV (Sarin et al., 2020; Tian et al., 2020; de Martel et al., 2020). There are several possible factors, including immunosuppressive drugs and anti-tumor treatments, activating the replication of HBV (Loomba and Liang, 2017; Shi and Zheng, 2020). HBV reactivation can cause several clinical complications, including mild liver dysfunction, liver failure in severe cases, and even death (Pattullo, 2016; Papatheodoridis et al., 2022). These complications usually cause the interruption of anti-tumor therapy. Therefore, monitoring HBV-related HCC patients for HBV reactivation is essential, especially during anti-tumor therapy.

Over the past few years, HBV reactivation has been observed after anti-tumor treatment, including interventional therapy, TKIs, and PD1s (Loomba and Liang, 2017; Shen et al., 2023). A retrospective study reported that HCC patients treated with TKIs plus PD1s could suffer HBV reactivation and combination therapy was a risk factor for reactivation of HBV (Lei et al., 2023). Another study revealed that HBV reactivation was more common with HAIC combined with PD1s than with PD1s monotherapy (He et al., 2021). However, no study has investigated the details of HBV reactivation and compared the survival of HBV-related HCC patients treated with HAIC and lenvatinib plus PD1s (HAIC-Len-PD1s) with and without HBV reactivation. This retrospective study aimed to investigate the HBV reactivation in HBV-related HCC patients treated with HAIC-Len-PD1s and assess the prognosis and adverse events (AEs).

## Materials and methods

### Patient recruitment and study design

**Figure 1:**
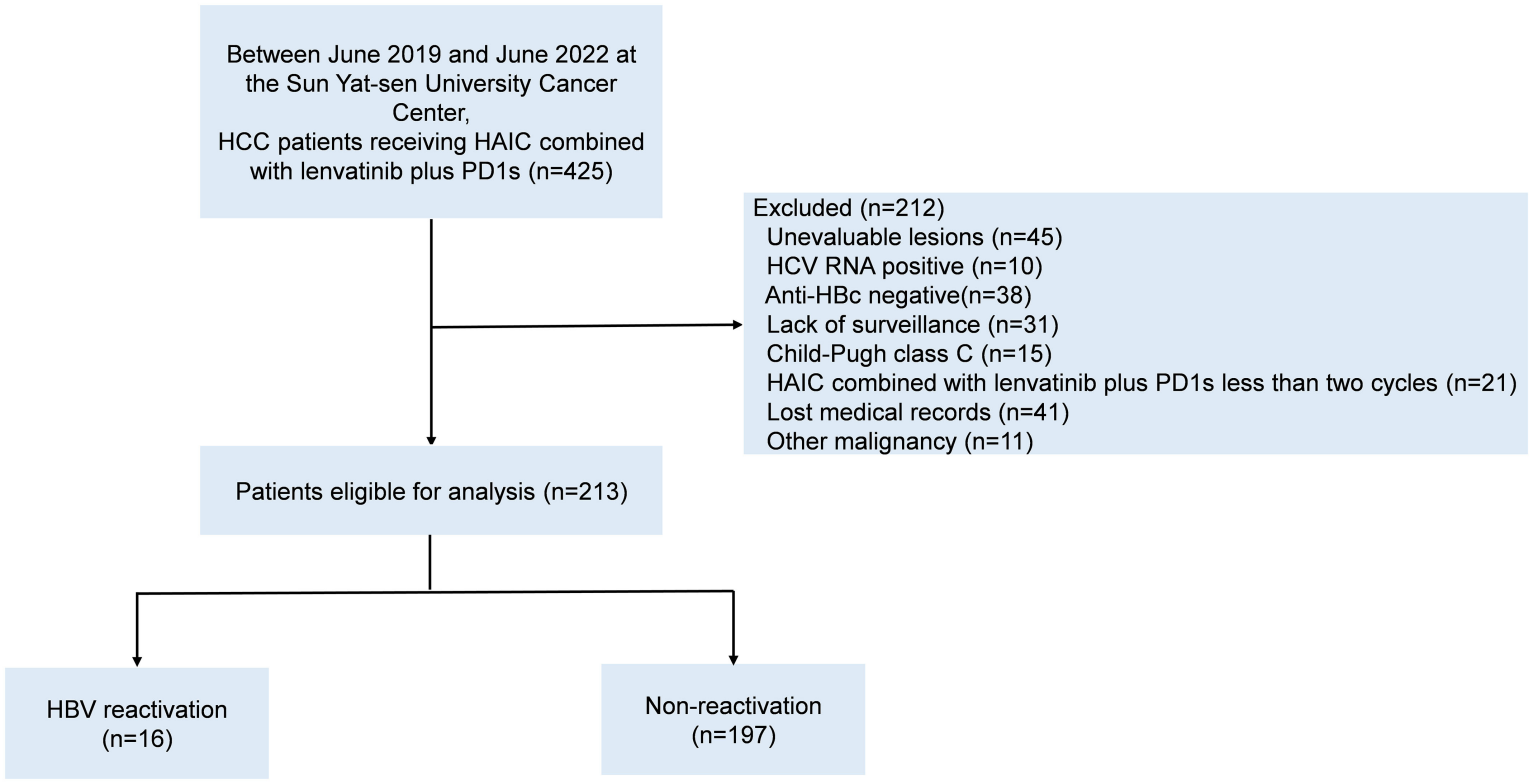
The patients’ flowchart. HCC, hepatocellular carcinoma; HAIC, hepatic arterial infusion chemotherapy; PD1s, programmed death receptor-1 signaling inhibitors; HCV, hepatitis C virus; Anti-HBc, antibody to hepatitis B coreantigen; HBV, hepatitis B virus.

We retrospectively collected 425 HBV-related HCC patients receiving HAIC-Len-PD1 at Sun Yat-sen University Cancer Center from June 2019 to June 2022. The inclusion criteria were as follows: (1) confirmed HCC diagnosis on histological examination, (2) chronic or past HBV infection (positive/negative hepatitis B surface antigen [HBsAg] and positive hepatitis B core antibody [HBcAb]); (3) received at least two cycles of HAIC-Len-PD1s, (4) at least one measurable intra-hepatic lesion; (5) Child–Pugh A or B class of liver function and (6) complete medical records. Patients with any other malignant tumor or other viral infections were excluded. Finally, 213 HBV-related HCC patients were included in the study (**Figure 1**). All patients were received HAIC-Len-PD1 as the first-line treatment.

### Treatment procedure

Patients underwent HAIC with 5−fluorouracil, oxaliplatin and leucovorin (FOLFOX) every 3 weeks combined with lenvatinib and PD1s at the initiation of treatment. The treatment plan of HAIC was performed according to our previously reported protocol (Li et al., 2022; Lyu et al., 2022). The HAIC treatment was repeated at 3-week intervals. Patients with body weight <60 kg received 8 mg lenvatinib once daily, while those ≥60 kg received 12 mg once daily. PD1s were administered intravenously at the recommended dose and were started on the same day when the HAIC ended. The interval between HAIC and PD1s treatment was less than a day. The PD1s used in this study included tislelizumab, camrelizumab, toripalimab, sintilimab, and pembrolizumab. Meanwhile, some patients were treated with antiviral therapy, and the antiviral drugs included entecavir (ETV, 0.5 mg/day), tenofovir disoproxil fumarate (TDF, 300mg/day), and tenofovir alafenamide fumarate (TAF, 25mg/day). Eighty patients received ETV, 45 patients received TDF, and 56 patients received TAF before HAIC-Len-PD1s.

### Data collection and outcomes

All baseline data were obtained from the medical records system of Sun Yat-sen University Cancer Center. Data included demographic characteristics, blood investigations, biochemical investigations, alpha-fetoprotein (AFP) level, HBV DNA level, and hepatitis B serologic test. The blood tests were conducted within 1 week before starting the treatment. HBV DNA level were conducted every 6 weeks to supervise HBV reactivation. Abdominal magnetic resonance imaging or computed tomography (CT), performed at baseline and every 6 weeks, was used to evaluate the radiological response. To confirm extra-hepatic metastasis, emission CT and positron emission tomography were performed.

The primary outcome was the risk of HBV reactivation. The Asian-Pacific Association for the Study of the Liver clinical practice guideline defines HBV reactivation in chronic HBsAg-positive patients as ≥2 log HBV DNA levels compared to baseline or >100 IU/mL in those with undetectable HBV DNA at baseline. Similarly, the guideline for reactivation in HBsAg-negative and anti-HBc-positive patients is HBsAg-negative becoming HBsAg-positive or undetectable HBV DNA becoming detectable HBV DNA (Lau et al., 2021). Fulfillment of any of the aforementioned criteria indicated HBV reactivation. The secondary outcomes were overall survival (OS), progression−free survival (PFS), and treatment−related AEs. The time interval from the start of HAIC-Len-PD1s to cancer-related death or the last follow-up was considered as OS. The interval from the start of treatment to disease progression, death, or the last follow-up was considered as PFS. Response Evaluation Criteria in Solid Tumors (RECIST, version 1.1) and HCC-specific modified RECIST (mRECIST) were used to assess the tumor response (Eisenhauer et al., 2009; Llovet and Lencioni, 2020). The tumor response was evaluated by two radiologists independently.

### Statistical analysis

The unpaired student’s t-test and Mann–Whitney U test were used to analyze continuous parametric variables and continuous non-parametric variables, respectively. Pearson’s chi-square test or Fisher’s exact probability test was used to analyze the categorical data. To identify the independent risk factors for HBV reactivation, univariate and multivariate logistic regression analyses using “Forward LR” were employed. Univariate regression was performed first, and parameters with P-values <0.1 that influenced HBV reactivation were taken into the multivariate analysis. Kaplan–Meier curves were used to represent the OS and PFS, and the log-rank test was used to analyze survival. *P*-values <0.05 were considered statistically significant. All analyses were performed using Statistical Product and Service Solutions (SPSS version 25.0, Inc., Chicago, USA) and R software (R version 4.1.1, R Foundation, Vienna, Austria).

## Results

### Patient characteristics

We enrolled 425 HCC patients receiving HAIC-Len-PD1s from June 2019 to June 2022, of which 213 patients met the inclusion criteria (**Figure 1**). **Table 1** shows the baseline characteristics of the included patients.

**Table 1:** Baseline characteristics of HCC patients.

Variables	Total (n=213)	HBV reactivation (n=16)	Non-reactivation (n=197)	*P* value
Age, years	51.4 ± 10.4	50.3 ± 11.5	51.5 ± 10.3	0.666
Sex				1.000
Male	194 (91.1)	15 (93.8)	179 (90.9)	
Female	19 (8.9)	1 (6.3)	18 (9.1)	
History of alcoholism				0.997
Yes	60 (28.2)	4 (25)	56 (28.4)	
No	153 (71.8)	12 (75)	141 (71.6)	
Antiviral prophylaxis				<0.001
Yes	181 (85)	9 (56.3)	172 (87.3)	
No	32 (15)	7 (43.7)	25 (12.7)	
HBsAg				1.000
Seropositive	190 (89.2)	14 (87.5)	176 (89.3)	
Seronegative	23 (10.8)	2 (12.5)	21 (10.7)	
HBeAg				0.605
Seropositive	44 (20.7)	2 (12.5)	42 (21.3)	
Seronegative	169 (79.3)	14 (87.5)	155 (78.7)	
ECOG PS				0.540
0	53 (24.9)	5 (31.3)	48 (24.4)	
1-2	160 (75.1)	11 (68.7)	149 (75.6)	
Child Pugh score				0.930
5	130 (61)	9 (56.3)	121(61.4)	
6	60 (28.2)	5 (31.3)	55 (27.9)	
7	18 (8.5)	2 (12.5)	16 (8.1)	
8	5 (2.3)	0	5 (2.6)	
ALBI grade				0.790
I	118 (55.4)	10 (62.5)	108 (54.8)	
II	93 (43.7)	6 (37.5)	87 (44.2)	
III	2 (0.9)	0	2 (1)	
Cirrhosis				0.972
Yes	134 (62.9)	10 (62.5)	124 (62.9)	
No	79 (37.1)	6 (37.5)	73 (37.1)	
Tumor diameter (cm)	11.3 ± 4.4	12.8 ± 3.1	11.1 ± 4.5	0.158
Tumor number				1.000
Single	37 (17.4)	3 (18.8)	34 (17.3)	
Multiple	176 (82.6)	13 (81.3)	163 (82.7)	
PVTT classification				0.604
Vp2	37 (17.4)	3 (18.8)	34 (17.3)	
Vp3	77 (36.1)	5 (31.3)	72 (36.5)	
Vp4	67 (31.5)	7 (43.8)	60 (30.5)	
No	32 (15)	1 (6.3)	31 (15.7)	
Extra−hepatic metastasis				0.981
Yes	147 (69)	11 (68.8)	136 (69)	
No	66 (31)	5 (31.3)	61 (31)	
HBV DNA, IU/ml				<0.001
Undetectable	67 (31.5)	12 (75)	55 (27.9)	
Detectable	146 (68.5)	4 (25)	142 (72.1)	
≥ 2000	84 (57.5)	0	84 (59.2)	0.031
<2000	62 (42.5)	4 (100)	58 (40.8)	
AFP, ng/ml	32045(0.9-121000)	27832 (46-121000)	2084 (0.9-121000)	0.166
ALT, IU/L	53.3 ± 41.2	50 ± 26.9	53.5 ± 42.2	0.745
AST, IU/L	99.3 ± 111.4	91.7 ± 75.8	99.9 ± 113.9	0.779
Albumin, g/L	40.8 ± 4.7	40.5 ± 5.7	40.9 ± 4.7	0.762
TBil, μmol/L	22.1 ± 26.4	18.5 ± 11.7	22.4 ± 27.2	0.571
WBC, ×10^9^/L	7.8 ± 3.6	8.2 ± 2.6	7.8 ± 3.7	0.621
Hemoglobin, g/L	142.6 ± 23.3	142.4 ± 20.6	142.6 ± 23.6	0.978
Platelet, ×109/L	251.5 ± 109.9	267 ± 73.8	250.2 ± 112.5	0.557
Cycles of HAIC	4(2-8)	4 (2-8)	4 (2-6)	0.927
PD1s category				0.765
Pembrolizumab	13 (6.1)	1 (6.3)	12 (6.1)	
Sintilimab	86 (40.4)	5 (31.3)	81 (41.1)	
Toripalimab	61 (28.6)	6 (37.5)	55 (27.9)	
Camrelizumab	10 (4.7)	0	10 (5.1)	
Tislelizumab	43 (20.2)	4 (25)	39 (19.8)	

HBV, hepatitis B virus; HBsAg, hepatitis B surface antigen; HBeAg, hepatitis B e antigen; ECOG PS Eastern Cooperative Oncology Group performance status; ALBI grade, Albumin-Bilirubin grade; PVTT, portal vein tumor thrombosis; DNA, deoxyribonucleic acid; AFP alpha−fetoprotein, ALT, alanine aminotransferase; AST, aspartate aminotransferase; TBil, total bilirubin; WBC, white blood cell; HAIC hepatic arterial infusion chemo−therapy; PD1s, programmed death receptor-1 inhibitors.

In total, 181 patients received antiviral prophylaxis before HAIC-Len-PD1s treatment. Most of the cases (89.2%) were HBsAg-positive, 146 patients (68.5%) had detectable HBV DNA levels, and 84 patients had HBV DNA levels >2000 IU/mL. Most of the patients (82.6%) had multiple tumors with a mean diameter of 11.3 ± 4.4 cm. The median frequency of the HAIC cycles was four (range 2–8).

### HBV reactivation

Among the 213 HBV-related HCC patients, 16 (7.5%) experienced HBV reactivation (HBV reactivation group), with a median interval time of 5.45 months (range 1.8–15 months) until reactivation (**Table 2**), while 197 (92.5%) did not (non-reactivation group). In the HBV reactivation group, 1 (6.2%) were female, and 4 (25%) had detectable HBV DNA levels before HAIC-Len-PD1s treatment. The median HBV DNA level at reactivation was 1070 IU/mL (range 80–118,000 IU/mL). Notably, two patients with HBsAg-negative results at baseline experienced HBV reactivation, HBV DNA levels became detectable, and HBsAg became positive (**Supplementary Table 1**). **Table 3** shows the risk of HBV reactivation based on antiviral prophylaxis and HBsAg status. HBV reactivation occurred in 14 (7.4%) patients out of 189 HBsAg-positive patients and 2 (8.3%) patients out of 24 HBsAg-negative and anti-HBc-positive patients. The incidence of HBV reactivation was 5% and 21.9% in patients with and without antiviral prophylaxis, respectively. There were significant differences in the antiviral prophylaxis (*P*<0.001) and HBV DNA levels (*P*<0.001) between the HBV reactivation and non-reactivation groups (**Table 1**).

**Table 2:** Details of 16 patients with HBV reactivation.

Patient characteristics			Baseline				At reactivation				
No.	Age/Sex	HAIC (frequency)	PD1s category	HBsAg	HBV DNA IU/ml	Antiviral prophylaxis	Intervals (months)	HBsAg	HBV DNA IU/ml	Antiviral treatment	Peak ALT, U/L
1	47/M	4	Pembrolizumab	(+)	Undetectable	ETV	3.5	(+)	238	ETV	39.5
2	49/M	4	Toripalimab	(+)	Undetectable	ETV	3.8	(+)	4400	ETV	46.1
3	50/M	4	Sintilimab	(+)	Undetectable	/	5	(+)	126	ETV	215.7
4	80/M	2	Sintilimab	(+)	80.4	ETV	7.5	(+)	4110	ETV	22.9
5	44/M	6	Toripalimab	(+)	132	ETV	4.1	(+)	7460	ETV	21
6	58/M	4	Tislelizumab	(+)	Undetectable	/	4.8	(+)	833	ETV	57.3
7	48/M	2	Sintilimab	(+)	Undetectable	/	2.5	(+)	2910	ETV	61.3
8	55/M	2	Tislelizumab	(+)	Undetectable	/	6.4	(+)	952	ETV	92.9
9	48/M	6	Tislelizumab	(+)	211	ETV	10.1	(+)	3470	ETV	28.2
10	45/F	4	Sintilimab	(-)	Undetectable	/	6.7	(+)	80	ETV	17.4
11	43/M	4	Tislelizumab	(-)	Undetectable	/	12.5	(+)	1150	ETV	47
12	65/M	4	Toripalimab	(+)	Undetectable	TDF	15.0	(+)	118000	TAF	146.8
13	43/M	4	Toripalimab	(+)	Undetectable	ETV	7.6	(+)	990	ETV	39.1
14	60/M	4	Sintilimab	(+)	Undetectable	ETV	2.8	(+)	515	ETV	54.6
15	30/M	4	Toripalimab	(+)	594	ETV	5.9	(+)	11000	ETV	36.3
16	40/M	6	Toripalimab	(+)	Undetectable	/	1.8	(+)	326	ETV	69

HBV, hepatitis B virus; HAIC hepatic arterial infusion chemo−therapy; HBsAg, hepatitis B surface antigen; DNA, deoxyribonucleic acid; ALT, alanine aminotransferase; M, male; F, female; ETV, entecavir; TAF, tenofovir alafenamide; TDF, tenofovir disoproxil fumarate.

**Table 3:** Incidences of HBV reactivation based on HBsAg status and antiviral prophylaxis.

	Total, n	HBV reactivation, n (%)
HBsAg-positive patients	189	14 (7.4)
HBsAg-negative and HBcAb-positive patients	24	2 (8.3)
No antiviral prophylaxis	32	7 (21.9)
Undetectable baseline HBV DNA	25	7 (28)
Detectable baseline HBV DNA	7	0
Antiviral prophylaxis	181	9 (5)
Undetectable baseline HBV DNA	42	5 (11.9)
Detectable baseline HBV DNA	139	4 (2.9)

HBV, hepatitis B virus; HBsAg, hepatitis B surface antigen; HBcAb, hepatitis B core antibody; DNA, deoxyribonucleic acid.

### HBsAg seroclearance

During the follow-up of 213 HBV-related HCC patients, only 2 (1%) patients showed HBsAg seroclearance. The detailed clinical characteristics of these patients were presented in **Supplementary Table 2**. They received ETV as prophylactic antiviral therapy with undetectable HBV DNA levels. Both patients continued ETV treatment until HBsAg seroclearance. The interval time until HBV reactivation of the two patients was 5.6 months and 29 months, respectively.

### Univariate and multivariable logistic regression analyses for HBV reactivation

Univariate and multivariate logistic regression analyses of the clinical characteristic parameters were performed to predict the risk factors associated with HBV reactivation (**Table 4**). The univariate analyses revealed that lack of antiviral prophylaxis (odds ratio [OR] 0.187, 95% confidence interval [CI] 0.064–0.547; *P*=0.002) and tumor diameter ≥10 cm (OR 5.312, 95% CI 1.176–24.005; *P*=0.03) were significant risk factors for HBV reactivation. Except for these two variables, all parameters with *P*-values <0.1, including AFP level and HAIC cycles, which could influence HBV reactivation, were included in the multivariate analysis. The multivariate logistic analysis revealed that lack of antiviral prophylaxis (OR 0.193, 95% CI 0.064–0.58; *P*=0.003) and tumor diameter ≥10 cm (OR 5.132, 95% CI 1.114–23.634; *P*=0.036) were significant and independent risk factors for HBV reactivation (**Table 4**).

**Table 4:** Univariate and multivariate logistic regression analysis for risk factors of HBV reactivation.

	Univariate		Multivariate	
	OR(95%CI)	*P* value	OR(95%CI)	*P* value
Age (≥50 years)	0.419 (0.146-1.199)	0.105		
Sex(male)	0.663 (0.083-5.314)	0.699		
History of alcoholism (yes)	0.839 (0.26-2.713)	0.77		
Antiviral prophylaxis (yes)	0.187 (0.064-0.547)	0.002	0.193 (0.064-0.58)	0.003
HBsAg (+)	0.835 (0.177-3.932)	0.82		
HBeAg (+)	0.527 (0.115-2.411)	0.409		
HBV DNA (>2000 IU/ml)	0	0.996		
Child Pugh score (B)	1.197 (0.254-5.636)	0.82		
Tumor diameter (≥10cm)	5.312 (1.176-24.005)	0.03	5.132 (1.114-23.634)	0.036
Tumor number (multiple)	0.904 (0.244-3.345)	0.88		
PVTT (≥2)	2.801 (0.357-21.984)	0.327		
Extra−hepatic metastasis (yes)	0.987 (0.329-2.963)	0.981		
AFP (≥400ng/ml)	3.026 (0.835-10.96)	0.092	2.379 (0.594-9.531)	0.221
ALT (≥50IU/L)	0.665 (0.222-1.987)	0.465		
Albumin (≥35 g/L)	0.573 (0.152-2.163)	0.411		
TBil (≥17.1μmol/L)	0.475 (0.166-1.357)	0.475		
WBC (≥11*10^9^/L)	1.663 (0.442-6.264)	0.452		
Cycles of HAIC (≥4)	2.963 (0.818-10.734)	0.098	2.074 (0.523-8.217)	0.299

HBV, hepatitis B virus; HBsAg, hepatitis B surface antigen; HBeAg, hepatitis B e antigen; DNA, deoxyribonucleic acid; PVTT, portal vein tumor thrombosis; AFP alpha−fetoprotein, ALT, alanine aminotransferase; TBil, total bilirubin; WBC, white blood cell; HAIC hepatic arterial infusion chemo−therapy.

### Patient prognosis and tumor response

**Figure 2:**
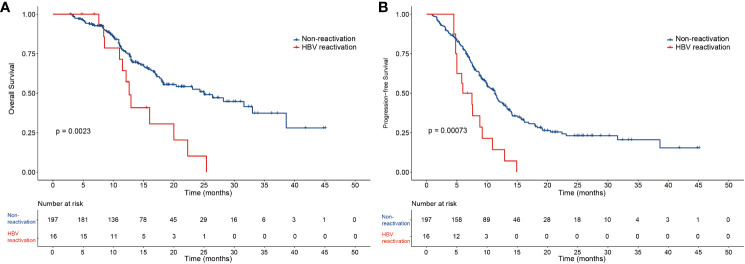
The overall survival and progression-free survival of the two groups of patients. Kaplan-Meier curves of **(A)** overall survival and **(B)** progression-free survival for patients in the HBV reactivation and non-reactivation groups.

The median OS and PFS of the 213 patients with HBV-related HCC were 22.3 months and 10.8 months, respectively (**Supplementary Figure 1**). In the HBV reactivation and non-reactivation groups, the median follow-up time was 20.0 months and 19.4 months, median OS was 12.6 months (95% CI 11.2–13.9) and 25 months (95% CI 16.8–27.7) (*P*=0.0023) (**Figure 2A**), and median PFS was 6.0 months (95% CI 3.0–9.0) and 11.3 months (95% CI 10–12.6) (*P*=0.00073), respectively (**Figure 2B**). Thus, patients in the non-reactivation group had a better prognosis than those in the HBV reactivation group. According to the mRECIST, the non-reactivation group patients had a better objective response rate (ORR) than the HBV reactivation group patients (65.6% vs. 31.3%; *P*=0.006). Furthermore, complete remission was observed in 18 (9.1%) patients and 1 (6.3%) patient in the non-reactivation and HBV reactivation groups, respectively. Partial response was observed in 111 (56.3%) patients and 4 (25%) patients in the non-reactivation and HBV reactivation groups, respectively. The details of the tumor response are summarized in **Supplementary Table 3**.

### AEs and safety

The treatment-related AEs are summarized in **Table 5**. Overall, the number of AEs was more in the HBV reactivation than in the non-reactivation group, especially hepatic AEs. Patients with HBV reactivation were more likely to have fever than those without HBV reactivation (*P*=0.002). In the HBV reactivation and non-reactivation groups, elevated alanine transaminase levels were observed in 7 patients and 40 patients (*P*=0.03), elevated aspartate aminotransferase levels in 11 patients and 80 patients (*P*=0.029), and hyperbilirubinemia in 12 patients and 82 patients (*P*=0.02), respectively. For these patients with abnormal liver function, we performed liver-protective therapy and symptomatic therapy actively. If necessary, the medication dose was adjusted, or the treatment was discontinued. The liver function returned to normal in most of the patients after treatment, and liver failure was not observed in any patient (**Supplementary Figure 2**). Moreover, all AEs were controlled, and no toxicity−related death was reported during the follow-up period.

**Table 5:** Objective treatment-related adverse events according to HBV reactivation.

Adverse events	Any Grade			Grade 3-4		
	HBV-reactivation (n=16)	Non-reactivation (n=197)	*P*	HBV-reactivation (n=16)	Non-reactivation (n=197)	*P*
Fever	5	15	0.002	0	0	NA
Fatigue	5	30	0.096	0	0	NA
Hypertension	1	5	0.938	0	0	NA
Cough	1	4	0.831	0	0	NA
Vomiting	5	35	0.184	0	0	NA
Diarrhea	1	6	1.000	0	0	NA
Abdominal pain	8	60	0.107	0	0	NA
Leukopenia	3	15	0.283	1	1	0.346
Anemia	7	53	0.15	0	3	1.000
Skin rash	0	15	0.61	0	1	1.000
Hypothyroidism	1	8	1.000	0	0	NA
Hepatic adverse events						
Elevated ALT	7	40	0.03	2	10	0.5
Elevated AST	11	80	0.029	2	13	0.705
Hypoalbuminemia	9	93	0.486	0	0	NA
Hyperbilirubinemia	12	82	0.02	1	0	1.000

HBV, hepatitis B virus; NA not applicable; ALT, alanine aminotransferase; AST, aspartate aminotransferase.

## Discussion

This retrospective study is the first to clarify the HBV reactivation in HBV-related HCC patients treated with HAIC-Len-PD1s and analyze the prognosis and AEs. In this study, 16 (7.5%) patients had HBV reactivation. The incidence of HBV reactivation was 5% and 21.9% in patients with and without antiviral prophylaxis, respectively. The OS and PFS were longer and AEs were lesser in the non-reactivation group than in the HBV reactivation group. Moreover, lack of antiviral prophylaxis and tumor diameter ≥10 cm were independent risk factors for HBV reactivation.

Previous studies have reported that anti-tumor treatments, including HAIC, TKIs, and PD1s, are associated with HBV reactivation. In this study, HBV reactivation was observed in 7.5% of patients. The incidence of HBV reactivation in this study was higher than that reported in other studies with patients treated with systemic therapy alone. A previous large cohort retrospective study reported that 0.5% of HCC patients showed HBV reactivation (Yoo et al., 2022). In the phase 3 IMbrave150 trial, the incidence of HBV reactivation was 2% and 7% in the atezolizumab plus bevacizumab arm and the sorafenib arm, respectively (Hsu et al., 2023). This could be because HAIC, a locoregional therapy, can increase the risk of viral reactivation. According to some studies, TACE, another locoregional therapy, is a risk factor for HBV reactivation, and the incidence of reactivation in patients treated with TACE was 10–35% (Shen et al., 2023; Jang, 2014). Another reason could be that combination treatment increases the risk of HBV reactivation. A recent retrospective study demonstrated that combination therapy was an independent risk factor for HBV reactivation (Lei et al., 2023).

In our study, HBV reactivation was reported in 9 (5%) and 7 (21.9%) patients with and without antiviral prophylaxis, respectively. Additionally, the lack of antiviral prophylaxis was an independent risk factor for HBV reactivation. These findings were consistent with those of several other studies. Over the past few years, HBV reactivation rates have reduced significantly because of the use of antiviral drugs. A study showed that in HBsAg-positive lymphoma patients treated with rituximab, the incidence of HBV reactivation reduced from 59.1 to 22.9% when they received prophylactic antiviral therapy (Hsu et al., 2014). Thus, HBV reactivation was more frequent in patients without antiviral prophylaxis than in those with antiviral prophylaxis, indicating the importance of antiviral prophylaxis. However, HBV reactivation occurred in some patients despite prophylactic antiviral therapy. This is a common phenomenon and has been reported in many studies (Shen et al., 2023; Jang, 2014; Hsu et al., 2014). This could be attributed to the development of resistance to antiviral therapy due to previous exposure to therapy (Tenney et al., 2009; Guo et al., 2018). Another possible reason could be that some patients received antiviral therapy irregularly due to unavoidable reasons, including missed medications and the unavailability of antiviral agents. Thus, HBV reactivation mostly occurs in patients without prophylactic antiviral therapy, and antiviral prophylaxis could effectively reduce the risk of viral reactivation.

According to the multivariate logistic regression analysis results, tumor diameter ≥10 cm was another independent risk factor. Several previous studies have shown that tumor diameter is a risk factor for HBV reactivation (Loomba and Liang, 2017; Lei et al., 2023). Notably, high baseline HBV DNA levels are usually associated with reactivation. However, in this study, no patient with HBV reactivation had a baseline HBV DNA level >2000 IU/mL. This could be because detection of HBV DNA is a routine examination for HCC patients, and once the serum HBV DNA level is above the normal value, effective antiviral treatment is implemented, which could prevent HBV reactivation.

Furthermore, two patients who were HBsAg-negative at baseline became HBsAg-positive after HAIC-Len-PD1s treatment. It was reported that an HBsAg-negative patient treated with immune checkpoint inhibitors became HBsAg-positive during HBV reactivation (Wong et al., 2021). This phenomenon could also be attributed to the reactivation of the HBV due to anti-tumor treatment. In our study, two patients showed HBsAg seroclearance when treated with antiviral therapy before and during HAIC-Len-PD1s treatment. Although the HBsAg seroclearance rate is low according to previous studies, administering antiviral therapy before and during the anti-tumor treatment could help achieve seroclearance.

The HBV DNA levels can affect the prognosis of HCC patients (Sun et al., 2021; Shen et al., 2021). Our study demonstrated that the non-reactivation group had longer PFS and OS than the HBV reactivation group. Therefore, an interactive relationship between tumor progression and HBV reactivation could be plausible. HBV reactivation results in the immune escape phase by activating several oncogenic signaling pathways and promotes HCC progression (Su et al., 2013). There are several kinds of immune cell subsets including exhausted HBV-specific CD8+ T cells, HBV-specific CD4+ T cells, and HBV-specific B Cells (Maini et al., 2000; Mason et al., 2016; Chen and Tian, 2019; Gehring and Protzer, 2019). Although the function of each cell population is different, they possibly promote the development of HCC together. First, HBV reactivation can increase the expression levels of TIM-3, suggesting that CD8+ T cells are functionally exhausted (Mohammadizad et al., 2019). Second, regulatory T cells are enriched and show greater expression of PD-1 with increased immunosuppression in HBV-related HCC patients (Lim et al., 2019). Finally, B cells of patients with HBV reactivation exhibit an atypical phenotype (CD21^−^ CD27^−^ B cells), which reduces antibody production (Salimzadeh et al., 2018; Burton et al., 2018). Thus, HBV reactivation can lead to a suppressive tumor immune microenvironment and promote tumor progression.

The mechanism of HBV reactivation in HCC patients receiving PD-1 inhibitors is unclear. We hypothesize that the reactivation of HBV may be attributed to the disruption of the immune environment, which is induced by PD-1 inhibitors. The administration of PD-1 inhibitors may lead to an excessive accumulation of unbound PD-1 and PDL-1 molecules in the body, which can exacerbate the condition of patients with HBV-related HCC. In cases where the PD-1 inhibitors fail to counteract the interaction between PD-1 and PDL-1 molecules, the PD-1/PD-L1 axis may be reactivated, resulting in the depletion of HBV-specific T cells with high expression of PD-1 molecules.

Our results clarified that AEs, especially hepatic AEs, were more frequent in patients with HBV reactivation than in those without reactivation. This could be because HBV reactivation can cause subsequent clinical complications, such as mild liver dysfunction. However, no toxicity−related death was observed during the follow-up period in our study. Moreover, these AEs were controllable, and the patients’ liver functions were restored after liver-protective treatment. Therefore, it is necessary to perform active intervention when HBV reactivation or even abnormal liver functions occur. Furthermore, this finding showed that the combination treatment of HAIC-Len-PD1s is relatively safe.

This study has certain limitations. First, it followed a single-center, retrospective design; hence, selection bias cannot be avoided. A prospective, multicenter, and randomized control trial is warranted to verify our conclusions. Second, mechanistic research was not conducted to clarify the association between HBV reactivation and HAIC-Len-PD1s treatment. Hence, further detailed bench-scale studies should be performed. Finally, the time intervals for HBV DNA or HBsAg screening displayed inconsistency both within and among the enrolled patients, and delayed testing may have contributed to unobserved endpoint events in some patients during the study period. A prospective, multicenter, and randomized control trial is warranted to verify our conclusions.

## Conclusion

This study demonstrated that treatment of HCC patients with HAIC-Len-PD1s could cause HBV reactivation. Patients with HBV reactivation had a worse prognosis than those without reactivation. Therefore, HBV-related HCC patients should undergo antiviral therapy and HBV DNA monitoring before and during the combination treatment.

## Data availability statement

The original contributions presented in the study are included in the article/**Supplementary Material**. Further inquiries can be directed to the corresponding authors.

## Ethics statement

The studies involving humans were approved by The Ethics Committee of Sun Yat-sen University Cancer Center. The studies were conducted in accordance with the local legislation and institutional requirements. The participants provided their written informed consent to participate in this study.

## Author contributions

ZY: Conceptualization, Data curation, Formal Analysis, Investigation, Methodology, Project administration, Software, Supervision, Validation, Writing – original draft. RG: Data curation, Investigation, Methodology, Software, Writing – original draft. YF: Data curation, Methodology, Project administration, Software, Writing – original draft. DH: Funding acquisition, Validation, Writing – review & editing. ZZ: Investigation, Validation, Writing – review & editing. MC: Conceptualization, Project administration, Resources, Validation, Visualization, Writing – review & editing. YZ: Conceptualization, Investigation, Project administration, Resources, Validation, Visualization, Writing – review & editing.

## Supplementary material

Supplementary Figure 1 The overall survival and progression-free survival of all patients. Kaplan-Meier curves of **(A)** overall survival and **(B)** progression-free survival for patients.

Supplementary Figure 2 The Sankey diagram of the dynamic changes of ALBI grades in the two groups of patients. The number above each bar represents the corresponding number of patients. HBV, hepatitis B virus; ALBI, Albumin-Bilirubin grade.
