# Supplementary figures and images for: Risk of hepatitis B virus reactivation and its effect on survival in advanced hepatocellular carcinoma patients treated with hepatic arterial infusion chemotherapy and lenvatinib plus programmed death receptor-1 inhibitors
Source: Front Cell Infect Microbiol. 2024 Feb 13;14:1336619. doi: 10.3389/fcimb.2024.1336619 (PMC10896825; doi:10.3389/fcimb.2024.1336619)

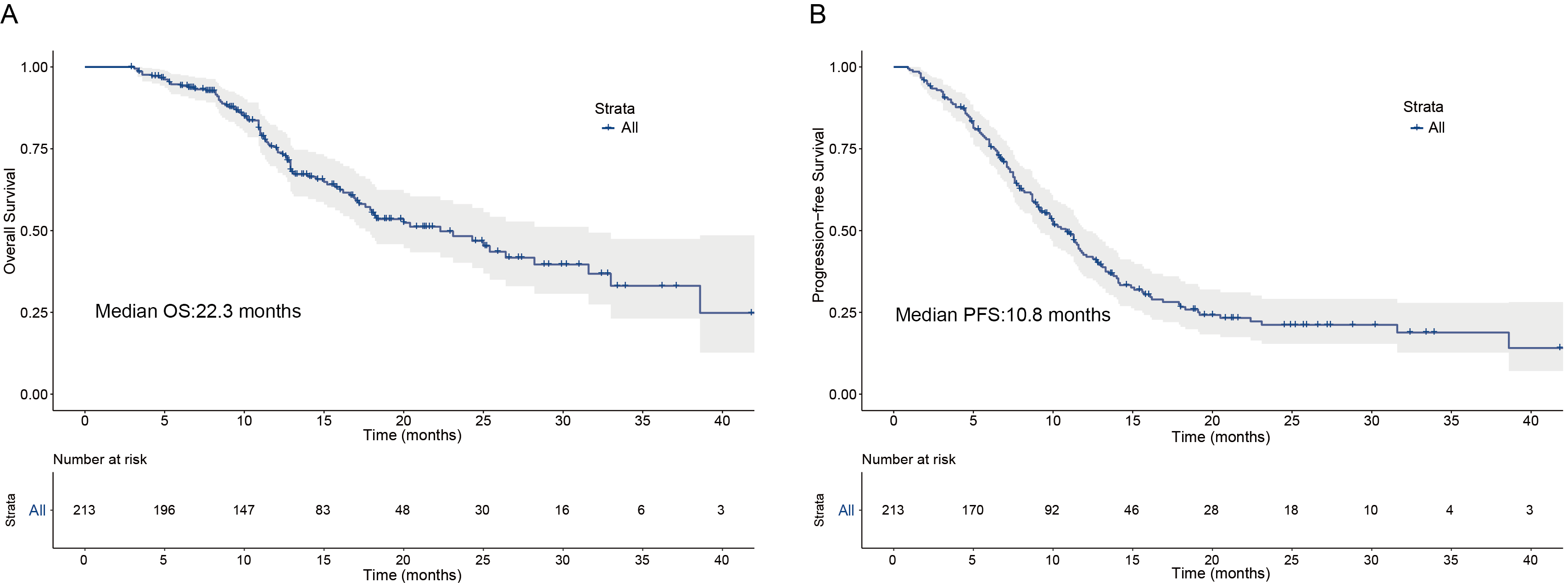

Supplement: Supplementary Figure 1 — The overall survival and progression-free survival of all patients. Kaplan-Meier curves of (A) overall survival and (B) progression-free survival for patients. [file Image_1.tif]

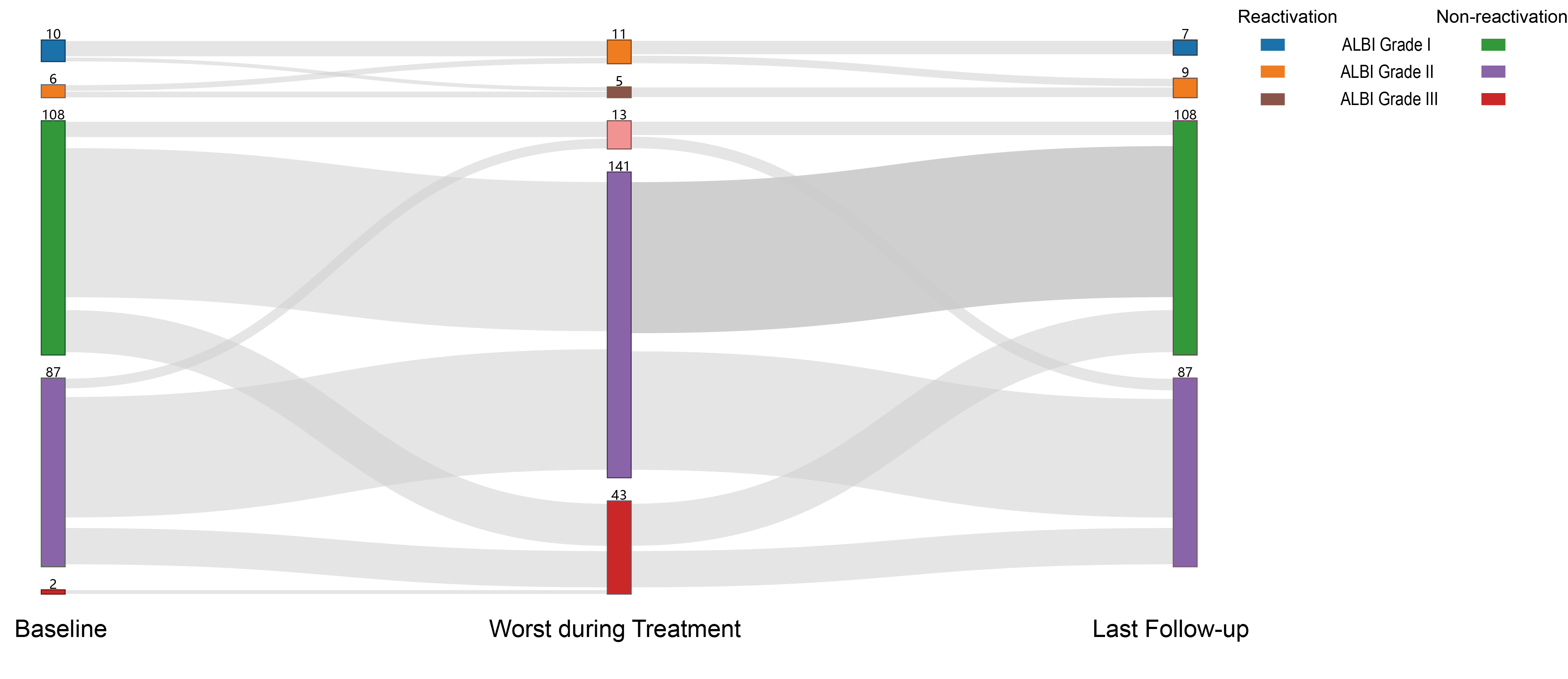

Supplement: Supplementary Figure 2 — The Sankey diagram of the dynamic changes of ALBI grades in the two groups of patients. The number above each bar represents the corresponding number of patients. HBV, hepatitis B virus; ALBI, Albumin-Bilirubin grade. [file Image_2.tif]
