# Supplementary material for: Risk of hepatitis B virus reactivation and its effect on survival in advanced hepatocellular carcinoma patients treated with hepatic arterial infusion chemotherapy and lenvatinib plus programmed death receptor-1 inhibitors
Source: Front Cell Infect Microbiol. 2024 Feb 13;14:1336619. doi: 10.3389/fcimb.2024.1336619 (PMC10896825; doi:10.3389/fcimb.2024.1336619)
Supplement: Supplementary file 3 [file DataSheet_1.docx]

**Supplementary Table 1:** Data of 2 baseline HBsAg-negative patients with HBV reactivation

| **Baseline** | | |
| --- | --- | --- |
| Age, years | 45 | 43 |
| Sex | F | M |
| Antiviral prophylaxis | No | No |
| HBsAg | (-) | (-) |
| HBeAg | (-) | (-) |
| HBV DNA, IU/ml | undetectable | undetectable |
| Child Pugh score | 5 | 5 |
| ALT, IU/L | 59.5 | 28 |
| Albumin, g/L | 40.1 | 47.4 |
| TBil, μmol/L | 19.4 | 17.2 |
| **At time of HBV reactivation** | | |
| Antiviral treatment | ETV | ETV |
| HBsAg | (+) | (+) |
| HBV DNA, IU/ml | 80 | 1150 |
| Interval, months | 6.7 | 12.5 |
| ALT, IU/L | 17.4 | 47 |
| Albumin, g/L | 33.2 | 48.4 |
| TBil, μmol/L | 11.8 | 13.7 |

**Abbreviations:** HBsAg, hepatitis B surface antigen; HBeAg, hepatitis B e antigen; HBV, hepatitis B virus; DNA, deoxyribonucleic acid; ALT, alanine aminotransferase; M, male; F, female; ETV, entecavir; TBil, total bilirubin.
