# Supplementary material for: Risk of hepatitis B virus reactivation and its effect on survival in advanced hepatocellular carcinoma patients treated with hepatic arterial infusion chemotherapy and lenvatinib plus programmed death receptor-1 inhibitors
Source: Front Cell Infect Microbiol. 2024 Feb 13;14:1336619. doi: 10.3389/fcimb.2024.1336619 (PMC10896825; doi:10.3389/fcimb.2024.1336619)
Supplement: Supplementary file 4 [file DataSheet_2.docx]

**Supplementary Table 2:** **Clinical characteristics of 2 patients who achieved HBsAg seroclearance**

| **Baseline** | | |
| --- | --- | --- |
| Age, years | 45 | 54 |
| Sex | F | M |
| Antiviral prophylaxis | ETV | ETV |
| HBsAg | (+) | (+) |
| HBeAg | (-) | (-) |
| HBV DNA, IU/ml | undetectable | undetectable |
| Child Pugh score | 5 | 7 |
| ALT, IU/L | 26.1 | 35.8 |
| Albumin, g/L | 44.3 | 47.2 |
| TBil, μmol/L | 8.1 | 59.7 |
| **At time of HBsAg seroclearance** | | |
| Antiviral treatment | ETV | ETV |
| HBsAg | (-) | (-) |
| HBV DNA, IU/ml | undetectable | undetectable |
| Interval, months | 5.6 | 29 |
| ALT, IU/L | 40.4 | 25.9 |
| Albumin, g/L | 35.6 | 32.6 |
| TBil, μmol/L | 30.1 | 25.9 |

**Abbreviations:** HBsAg, hepatitis B surface antigen; HBeAg, hepatitis B e antigen; HBV, hepatitis B virus; DNA, deoxyribonucleic acid; ALT, alanine aminotransferase; M, male; F, female; ETV, entecavir; TBil, total bilirubin.
