# Supplementary material for: Risk of hepatitis B virus reactivation and its effect on survival in advanced hepatocellular carcinoma patients treated with hepatic arterial infusion chemotherapy and lenvatinib plus programmed death receptor-1 inhibitors
Source: Front Cell Infect Microbiol. 2024 Feb 13;14:1336619. doi: 10.3389/fcimb.2024.1336619 (PMC10896825; doi:10.3389/fcimb.2024.1336619)
Supplement: Supplementary file 5 [file DataSheet_3.docx]

**Supplementary Table 3: Tumor Responses Evaluated by RECIST1.1 and mRECIST Criteria**

| **Response** | **RECIST1.1** | | | **mRECIST** | | |
| --- | --- | --- | --- | --- | --- | --- |
|  | **HBV reactivation**  **(n=16)** | **Non-reactivation**  **(n=197)** | ***P* value** | **HBV reactivation**  **(n=16)** | **Non-reactivation**  **(n=197)** | ***P* value** |
| CR | 0 | 2 (1%) | - | 1 (6.3%) | 18 (9.1%) | - |
| PR | 4 (25%) | 122 (61.9%) | - | 4 (25%) | 111 (56.3%) | - |
| SD | 9 (56.3%) | 46 (23.4%) | - | 8 (50%) | 41 (20.8%) | - |
| PD | 3 (18.8%) | 27 (13.7%) | - | 3 (18.8%) | 27 (13.7%) | - |
| ORR | 4 (25%) | 124 (62.9%) | 0.007 | 5 (31.3%) | 129 (65.6%) | 0.006 |
| DCR | 13 (81.3%) | 170 (86.3%) | 0.854 | 13 (81.3%) | 170 (86.3%) | 0.854 |

**Abbreviations:** HBV, hepatitis B virus; CR, complete response; PR, partial response; SD, stable disease; PD, progressive disease; ORR, objective response rate; DCR, disease control rate.
